# Reducing the proportion of *Pinus tabuliformis* planted in mixed coniferous-broad forest stabilizes the microbiological community composition

**DOI:** 10.3389/fpls.2025.1646980

**Published:** 2025-09-15

**Authors:** Zixing Li, Ran Wang, Runmei Gao, Zhenhua Hu, Mengtao Zhang

**Affiliations:** College of Forestry, Shanxi Agricultural University, Taigu, Shanxi, China

**Keywords:** *Pinus tabuliformis*, soil properties, soil microbiological community composition, soil bacteria, soil fungi

## Abstract

**Background:**

*Piuns tabuliformis* is an evergreen conifer species in North China and plays an important role in maintaining ecological security in North China. Soil microbiological, which are susceptible to disturbance by various external factors, is an important indicator of soil health and are crucial for maintaining soil biodiversity and ensuring the stability of ecosystem functions. However, the mechanisms driving changes in soil properties and soil microbiological community composition under different percentages of *P. tabuliformis* plantations are still poorly understood.

**Methods:**

In this study, we investigated soil properties and microbiological community composition in four forests with different percentages of *P. tabuliformis* plantation (PT10%, PT20%, PT60% and PT100%) in Zhongtiao Mountain.

**Results:**

The results showed that lowering the planting ratio of *P. tabuliformis* in mixed conifer-broad forests could improve soil pH and increase the nutrient reserves in the soil. The relative abundance of Ascomycota in the soil increased with the reduction of the planting proportion of *P. tabuliformis*. Co-occurrence network analyses showed that soil microbiological community composition was more stable in forests with lower percentages of *P. tabuliformis* plantings (PT20%).

**Conclusion:**

This study showed that soil microbiological community composition was more stable in forests with a low percentage of *P. tabuliformis* planting and a rich forest canopy of tree species. The planting proportion of *P. tabuliformis* is an important factor influencing soil microbiological community composition, which provides a new theoretical basis for rational management of mixed coniferous and broadleaf forests in warm-temperate continental climate zones.

## 1 Introduction

Forests are integral to terrestrial ecosystems, serving as crucial reservoirs of biodiversity and ecosystem services that support human well-being, conserve soil and water, mitigate pollution, and regulate ecological balance (Douh et al., 2018; Halofsky et al., 2018). Forest ecosystems store about half of the Earth’s terrestrial carbon and play an important role in the global carbon cycle (Xue et al., 2022). Soil is the most important habitat for microorganisms, which are diverse and multifunctional (Zhang et al., 2024c). Among these services, soil microorganisms are pivotal, bridging above- and below-ground processes through the soil carbon cycle (Liu et al., 2017), thereby maintaining biodiversity and ecosystem function (Rillig et al., 2019).

*Pinus tabuliformis* is an evergreen conifer species endemic to China, with a wide natural distribution area that extends as far north as the Yinshan Mountains in southern Liaoning and the Inner Mongolia Autonomous Region, and as far south as Sichuan Province and Chongqing Municipality. *P. tabuliformis* is the second largest in Pinus, and is the fourth largest forest cover type in China (Guo et al., 2008). *P. tabuliformis* can grow in a wide range of soil types (acidic, neutral and calcareous) (Guo et al., 2008), and is an important afforestation species for maintaining regional ecological security in North China, as well as an advantageous tree species in the Zhongtiao Mountains, with significant economic advantages (Ning et al., 2023).

Soil microbiological, which are susceptible to disturbance by various external factors, is an important indicator of soil health and are crucial for maintaining soil biodiversity and ensuring the stability of ecosystem functions. Soil microbiological diversity makes an important contribution to global biodiversity. Bacteria and fungi, as the most important soil microbiological, are reported to play different roles in ecosystem functions in which soil microorganisms are involved (Liu et al., 2023b). It has been shown that bacteria are one of the most abundant microorganisms in forest soils (Louca et al., 2016) and are able to efficiently degrade a wide range of apoplastic materials in forest ecosystems (Paterson et al., 2008). Soil fungi are able to break down lignin and cellulose in withered *P. tabuliformis* (Bahram et al., 2018). In addition, changes in canopy species diversity in forests can also cause changes in soil microbiological community composition.

The metabolic strategies of microorganisms are complex and diverse (Shaffer et al., 2022; Long et al., 2025), and the metabolic processes of microorganisms involve many biochemical reactions (Malik et al., 2018). Soil microorganisms decompose organic matter (Bardgett and van der Putten, 2014; Cheng et al., 2023), fueling microbiological activity and driving nutrient cycling (Liu et al., 2017). A global meta-analysis showed that afforestation can increase bacterial and fungal biomass (Luo et al., 2023). Changes in canopy plant species in forests affect soil microbiological community composition. Dominant tree species in forest ecosystems are reported to be important factors influencing the composition of soil microbiological communities (Urbanová et al., 2015). SOC is key factor driving microbiological community composition (Bai et al., 2019; Wan et al., 2024), and SOC content has been reported to be related to soil microbiological community composition (Lange et al., 2015; Tardy et al., 2015). A past study has shown that biomass carbon stocks in forests are generally larger than in other ecosystems under similar climatic conditions (Arevalo et al., 2009). A proper pH can put more energy into the growth and reproduction process of microorganisms (Long et al., 2025). Previous studies have shown that soil bacterial communities can be shaped by soil pH (Xue et al., 2017). Soil fungi are able to adapt to low soil pH and are extremely important to forest ecosystems (Midgley and Phillips, 2019). A study on *P. tabuliformis* showed that soils under *P. tabuliformis* forests are relatively poor and low in active N content (Zhang et al., 2019). Another study showed that the response of soil bacteria and fungi to N deposition varied depending on the climatic zone in which the forest was located. Forests in subtropical zones had more pronounced changes in soil fungi in response to N deposition compared to bacteria (Xue et al., 2025), but forests in temperate zones had higher soil fungal diversity with increasing soil N content (Liu et al., 2023a).

Currently, most studies on soil properties and soil microbes in forest ecosystems have focused on forest regeneration modes (Cheng et al., 2023), forest ecotypes (Li et al., 2021), forest restoration strategy years (Bai et al., 2019; Wang et al., 2024) or forest land management (Liu et al., 2022). In recent years, in order to increase the proportion of living trees and the proportion of large diameter trees, the Zhongcun Forestry has adopted target tree management measures based on the concept of near-natural management. Select high quality trees with a diameter of 15–30 cm at breast height as target trees (trees in the same canopy or upper canopy as the target trees and which will affect the growth of the target trees are considered as interference trees), and regularly cut down the interference trees that overlap with the canopy of the target trees, so as to realize the adjustment of the spatial structure of the forest stand and improve the quality of the soil. However, the changes in soil nutrients and soil microbiological community composition after target tree management are unknown, and the differences in soil properties and soil microbiological community composition in forests with different percentages of *P. tabuliformis* plantings have not been clearly concluded. Therefore, in order to determine the rational management mode of mixed conifer forest ecosystems in warm-temperate continental climate zones, it is necessary to gain an in-depth understanding of soil properties and soil microbiological communities following changes in the percentage of *P. tabuliformis* plantings. This study aimed to elucidate the differences in soil properties and soil microbiological community composition in mixed conifer forests planted with different proportions of *P. tabuliformis* in the Zhongtiao Mountain, and to analyze the reasons for these differences and the relationship between soil properties and soil microbiological community composition. We hypothesized that (i) lowering the proportion of planted *P. tabuliformis* in mixed coniferous-broad forest reduces soil acidity and increases soil nutrient storage; (ii) soil microbiological community is more stable in forests with low *P. tabuliformis* planting proportions; and (iii) different soil characteristics have direct and indirect effects on the composition of soil bacteria and fungi.

## 2 Materials and methods

### 2.1 Study site

The study site was located in Zhongtiao Mountain (Latitude 35°24′00″ to 35°40′00″ N, Longitude 111°56′12″ to 112°14′00″ E). Zhongtiao Mountain spans the cities of Linfen, Jincheng and Yuncheng, belongs to the tributaries of Taihang Mountain, and is located in the warm temperate semi-humid continental monsoon climate zone. The average annual temperature is 10.3°C, the lowest temperature is -8.2°C, the highest temperature is 28.8°C (Ning et al., 2023). The annual sunshine are 2,679.8 hours and the frost-free period is 197 days. The average annual precipitation is 600–720 mm, mainly in June to September. Soil types range from meadow soil to brown forest soil, drenched brown soil, and brown soil. The dominant tree species including *P. tabuliformis*, *Quercus mongolica*, *Betula platyphylla, Populus davidiana*, *Carpinus turczaninovii* and *Toxicodendron vernicifluum*.

**Figure 1:**
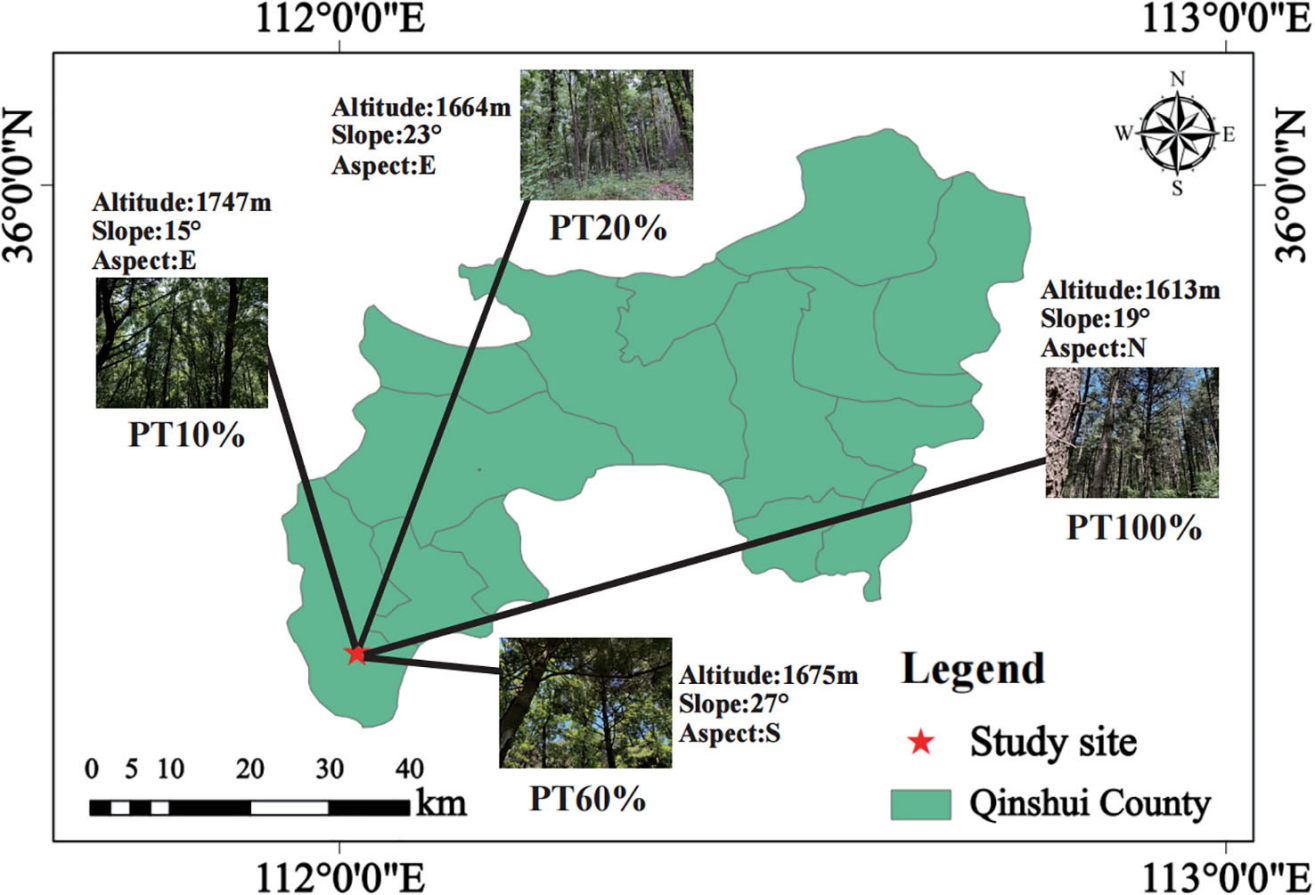
Location of the study area and photographs of sample plots of the four forests.

In July 2024, based on the proportion of *P. tabuliformis* (PT) in the forest, four sample plots with different percentages of *P. tabuliformis* planting were established in the Xiachuan Management Area of Zhongcun Forest Field under the similar standing conditions (10% *P. tabuliformis* planting, PT10%; 20% *P. tabuliformis* planting, PT20%; 60% *P. tabuliformis* planting, PT60%; 100% *P. tabuliformis* planting, PT100%), with four replicates for each sample plot. Each plot measured 20 m × 20 m. The location of the survey sample plots was recorded (**Figure 1**; **Supplementary Figure S1**). Tree species, diameter at breast height (DBH) and height of all trees with DBH ≥ 5 cm were recorded. Five 5 m × 5 m subplots were established within each 20 m × 20 m plot following the five-point sampling method to assess understory shrubs. A total of 80 subplots (5 × 16) were surveyed. At the center of each 5 m × 5 m subplot, a 1 m × 1 m subplot was used to investigate understory herbs. Shrub and herb species, ground diameter and height in the plots were recorded, and the top three dominant species in each plot were filtered by calculating their importance values (**Table 1**; **Supplementary Table S1**).

**Table 1:** Basic conditions of sample plots in different percentages of *P. tabuliformis* planting forests.

Sample plot type	Dominant tree species	Dominant shrub species	Dominant herb species	Mean DBH/cm	Mean height/m	Density/n m^-2^
PT10%	*Piuns tabuliformis*, *Quercus mongolica*, *Populus davidiana*, *Betula platyphylla*, *Carpinus turczaninovii*	*Forsythia suspensa*, *Campylotropis macrocarpa*, *Spiraea trilobata*	*Carex lancifolia*, *Melampyrum roseum*, *Stipa capillata*	12.5	13.8	0.11
PT20%	*Piuns tabuliformis*, *Quercus mongolica*, *Toxicodendron vernicifluum*, *Betula platyphylla*, *Carpinus turczaninovii*	*Forsythia suspensa*, *Cotoneaster multiflorus*, *Smilax stans*	*Carex lancifolia*, *Clematis florida*, *Phlomoides umbrosa*	16.0	10.9	0.11
PT60%	*Piuns tabuliformis*, *Quercus mongolica*	*Cotoneaster multiflorus*, *Forsythia suspensa*, *Spiraea ouensanensis*	*Carex lancifolia*, *Melampyrum roseum*, *Stipa capillata*	21.6	15.2	0.07
PT100%	*Piuns tabuliformis*	*Forsythia suspensa*, *Rubus sachalinensis*, *Philadelphus incanu*	*Carex lancifolia*, *Phlomoides umbrosa*, *Trichosanthes kirilowii*	23.7	16.6	0.08

The importance values (IV) of shrubs and herbs were calculated using the following formulas (Zhang et al., 2024b):


IV of shrub and herb=(relative density+relative dominance+relative coverage)/3

### 2.2 Soil sample collection

An additional 1 m × 1 m plot was set up in the center of each 5 m × 5 m subplots set up within each 20 m × 20 m plot, and soil samples were collected from the 1 m × 1 m plots. Three bags of 0–20 cm topsoil were collected from each sampling site, with 15 samples from each plot. In the laboratory, the 15 samples from each plot were combined, large stones and rhizomes were removed, and the mixture was sieved through a 0.9 mm sieve. The sieved samples were stored at -80°C for further analysis.

### 2.3 Soil sample determination and analysis

#### 2.3.1 Determination of soil properties

Soil pH was measured using a glass electrode with a soil suspension water to soil ratio of 1:2.5. Available potassium (AK) was extracted with a neutral 1 M NH4Ac solution and the content was subsequently determined by alkaline hydrolysis diffusion and flame photometry (Zhang et al., 2024a). Total nitrogen (TN) was measured using Kjeldahl nitrogen determination (Joergensen and Meyer, 2006). Microbial biomass nitrogen (MBN) and microbial biomass carbon (MBC) content were determined via chloroform fumigation (Vance et al., 1987; Joergensen and Meyer, 2006). Soil organic carbon (SOC) content was analyzed via the H_2_SO_4_-K_2_Cr_2_O_7_ oxidative technique (Nelson and Sommers, 1996).

#### 2.3.2 Soil DNA extraction, bacterial 16S and fungal ITS gene amplification, and sequencing

Total DNA was extracted from soil samples using OMEGA Soil DNA Kit (M5635-02, Omega Bio-Tek, Norcross, GA, USA). The bacterial 16S rRNA V3–V4 region was amplified with primers 338F (5’-barcode+ACTCCTACGGGAGGCAGCA-3’) and 806R (5’-GGACTACHVGGGTWTCTAAT-3’) (Mori et al., 2013), while fungal ITS regions were amplified with ITS5 (5’-GGAAGTAAAAGTCGTAACAAGG-3’) and ITS2 (5’-GCTGCGTTCTTCATCGATGC-3’) primers (Xiong et al., 2014). The PCR amplification cycles involved pre-denaturation at 98°C for 5 min, followed by 25 cycles of denaturation at 98°C for 30 s, annealing at 52°C for 30 s, extension at 72°C for 45 s, and a final extension at 72°C for 5 min. After the PCR amplification cycle, the PCR products were verified by 2% agarose gel electrophoresis, and the PCR products were quantified using the BioTek Microplate Reader (FLx800) and the Quant-iT PicoGreen dsDNA Assay Kit.

### 2.4 Statistical analysis

Statistical analyses were conducted in R (v4.4.1). Data were assessed for normality and homoscedasticity using normal distribution and mean square tests. One-way ANOVA followed by Least Significant Difference (LSD) multiple comparisons was used to analyze significant differences in soil properties between forests planted with different percentages of *P. tabuliformis*.

β-diversity of soil microbiological was analyzed using non-metric multidimensional scaling (NMDS) based on Bray-Curtis dissimilarity (Mei et al., 2025). PERMANOVA was applied to assess the influence of the proportion of planting of *P. tabuliformis* on the composition of soil microbiological, while analysis of similarity (ANOSIM) was used to evaluate differences between forests with different proportions of planting of *P. tabuliformis* (*n* = 999 permutations). The first axis of NMDS explains the greatest variation in microbiological community composition. Spearman’s correlation analysis was conducted to investigate symbiosis patterns among microbiological community composition in forests with four proportions of *P. tabuliformis* plantations, focusing on microorganisms with a relative abundance > 0.5% (Barberan et al., 2012). The Spearman’s correlation coefficient (r) > 0.6 with corresponding *p*-values< 0.01 were considered statistically robust and were used to generate the network. Results of the symbiotic network analysis were visualized using Gephi 0.10.1 (Bastian et al., 2009), which also identified dominant phyla. The colors of the nodes represent the dominant phyla. Network edges represented nodes that were pairwise related, with pink lines representing positive connections and green lines representing negative connections.

Spearman correlation analysis was used to study the relationship between soil properties and microbiological relative abundance of dominant phyla. Relationships between soil properties (pH, AK, TN, MBN, MBC and SOC) and the first NMDS axis (NMDS1) of soil microbiological diversity were explored using regression analysis (Liu et al., 2022). In order to test whether there are differences in the response of soil microorganisms to soil properties, the drivers of soil bacterial and fungal diversity were analyzed using the GLMMS. We fitted a model with soil microbiological diversity as the response variable. Sample plots with the same percentage of *P. tabuliformis* planting were considered as random effects. To ensure a uniform dimension, we standardized and centered the independent variables. To avoid collinearity, we grouped the independent variables (fixed effects) according to a hierarchical structure through hierarchical partitioning and further decomposed the degree of contribution of each independent variable to the dependent variable. Among the independent variables were divided into four groups, namely soil C (MBC and SOC), soil N (MBN and TN), AK and pH (Gao et al., 2024).

Partial Least Squares Passage Modeling (PLS-PM) (Tenenhaus et al., 2005) was conducted to explore the relationships among soil microbiological diversity (scores of the first NMDS axes for soil bacteria and fungi) (Cheng et al., 2023), percentage of *P. tabuliformis* planted in forests (percentage of PT), soil C (SOC, MBC), soil N (MBN, TN), AK and pH. PLS-PM is suitable for small sample sizes or not normally distributed data and contains both external and internal models. PLS-PM visualizes the results of the path analysis in the form of path coefficients, which are used to test the direction and strength of the causal relationships between latent variables. The fit of PLS-PM is represented by the coefficient of determination (R^2^) and the goodness of fit (GoF). The fit is weak when R^2^ is above 0.19, moderate when R^2^ is above 0.33, and strong when R^2^ is above 0.67. The goodness of fit is weak when GoF is above 0.1, moderate when GoF is above 0.25, and strong when GoF is above 0.36. This approach analyzed potential direct and indirect effects among these factors and the soil microbiological diversity.

## 3 Results

### 3.1 Soil properties

With the exception of MBC and SOC, all of the soil properties showed a decrease with increasing percentage of plantings of *P. tabuliformis*. Soil pH (*p* = 0.0024) in PT10% was significantly higher than in the other forests and was closest to neutral. Soil AK (*p* = 0.0013) was significantly higher in PT10% than in PT60% and PT100%, there were no significant differences between PT20% and PT60%, and soil AK content in PT20% was significantly higher than in PT100%. Soil TN (*p* = 0.0187) was significantly higher in PT10% than in PT100%. Soil MBN (*p* = 0.0027) was significantly higher in PT10% than in PT60% and PT100%. Soil MBC (*p* = 0.0008) and SOC (*p* = 0.0003) were significantly lower in PT100% than in the other three forests (**Table 2**).

**Table 2:** One-way ANOVA for forest soil properties with different percentages of *P. tabuliformis* planting.

Soil characteristics	PT10%	PT20%	PT60%	PT100%
pH	6.67 ± 0.35 a	5.97 ± 0.32 b	5.75 ± 0.23 b	5.55 ± 0.39 b
AK (mg·kg^-1^)	232.70 ± 37.63 a	216.08 ± 41.95 ab	162.30 ± 9.64 bc	133.72 ± 9.38 c
TN (g·kg^-1^)	3.61 ± 0.18 a	3.32 ± 0.23 ab	3.12 ± 0.51 ab	2.46 ± 0.66 b
MBN (mg·kg^-1^)	103.19 ± 4.69 a	82.39 ± 26.18 ab	58.57 ± 5.12 b	46.35 ± 21.78 b
MBC (mg·kg^-1^)	931.91 ± 12.69 a	962.48 ± 200.47 a	860.99 ± 28.61 a	551.02 ± 90.54 b
SOC (g·kg^-1^)	60.17 ± 2.98 a	53.10 ± 7.13 a	59.88 ± 5.33 a	35.11 ± 1.69 b

Values are mean ± standard deviation (n = 3). Values within the same rows followed by different letters are significantly different (*p*< 0.05).

### 3.2 Soil microbiological community composition and β-diversity

**Figure 2:**
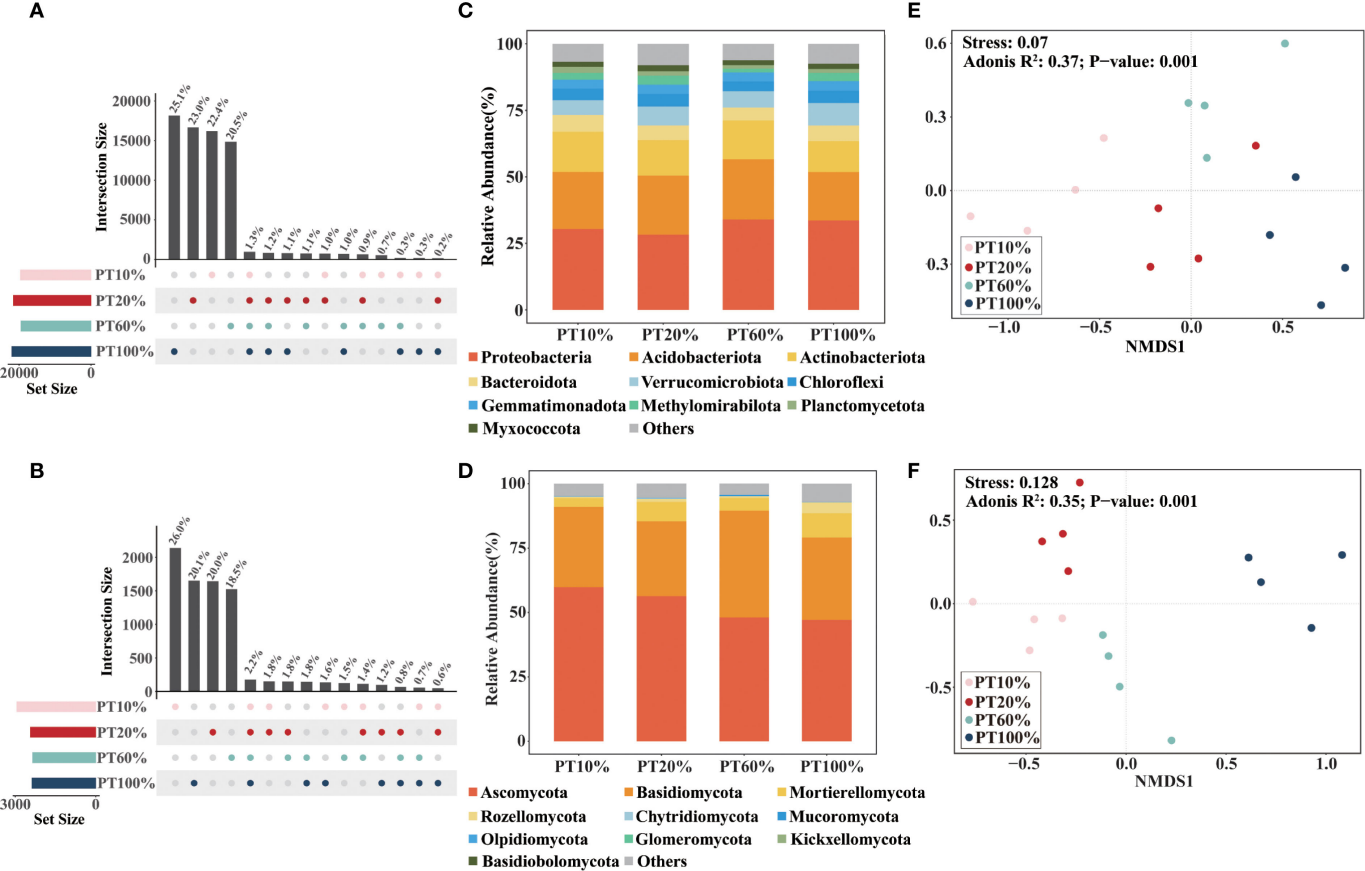
Soil microbiological community composition and β-diversity in forests with different percentages of *Pinus tabuliformi* plantings. **(A, B)** are the numbers of common and unique bacterial and fungal species, respectively. **(C, D)** are the relative abundance of dominant soil bacterial and fungal communities at the phylum classification, respectively. **(E, F)** are the non-metric multidimensional scaling (NMDS) plots of bacterial and fungal communities based on the Bray-Curtis distance, respectively.

Unique bacterial and fungal species were more prevalent than shared species across four different forests. Unique bacteria accounted for 91%, and unique fungi accounted for 84% of total taxa (**Figures 2A, B**). Proteobacteria, Acidobacteriota and Actinobacteriota were the dominant bacterial taxa, representing over 70% of sequences (**Figure 2C**). Dominant fungal taxa included Ascomycota, Basidiomycota, and Mortierellomycota, comprising more than 85% of sequences (**Figure 2D**). The relative abundance of Ascomycota in the soil decreased gradually with increasing percentage of *P. tabuliformis* planted. Soil microbiological β-diversity, assessed at the OTU level via NMDS based on Bray-Curtis distances, revealed significant compositional differences in soil bacteria (Stress = 0.070) (**Figure 2E**) and fungi (Stress= 0.128) (**Figure 2F**) among four different forests.

**Figure 3:**
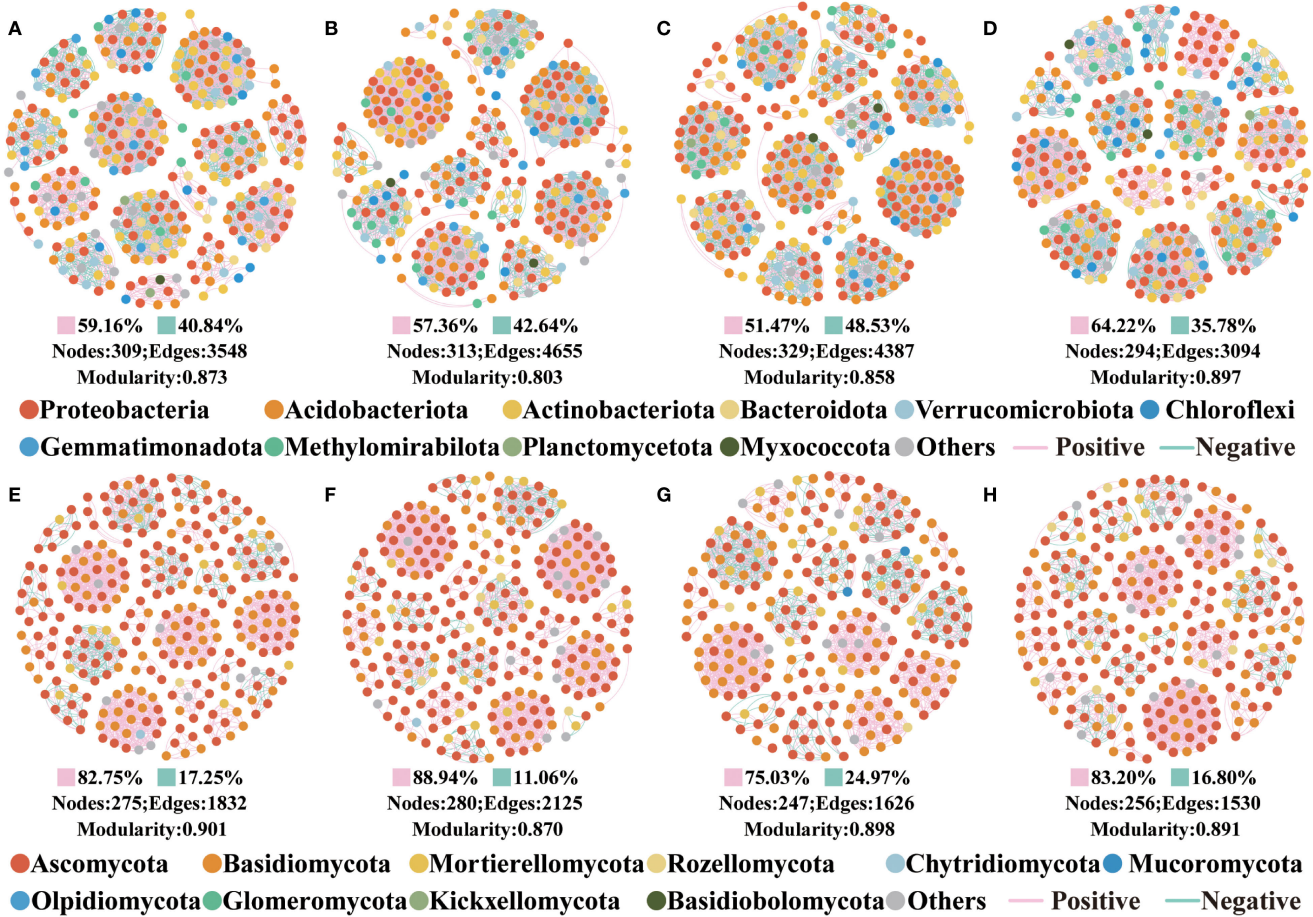
The co-occurrence networks in different forest types. **(A–D)** are the soil bacterial co-occurrence networks in PT10%, PT20%, PT60% and PT100%, respectively. **(E–H)** are the soil fungal co-occurrence networks in PT10%, PT20%, PT60% and PT100%, respectively. Pink edges indicate positive correlation, and green edges indicate negative correlation. Nodes are colored according to phyla.

Co-occurrence network topologies revealed that bacterial and fungal interactions varied by four forests (**Figure 3**). Overall, the complexity of the bacterial co-occurrence network was greater than that of the fungal. For bacteria, PT60% has the most complex network, PT20% the next most complex, and PT60% has the most nodes and edges. For fungi, PT20% has the most complex network, followed by PT10%, and PT20% has the most nodes and edges.

### 3.3 Relationship between soil properties and soil microbiological community composition

**Figure 4:**
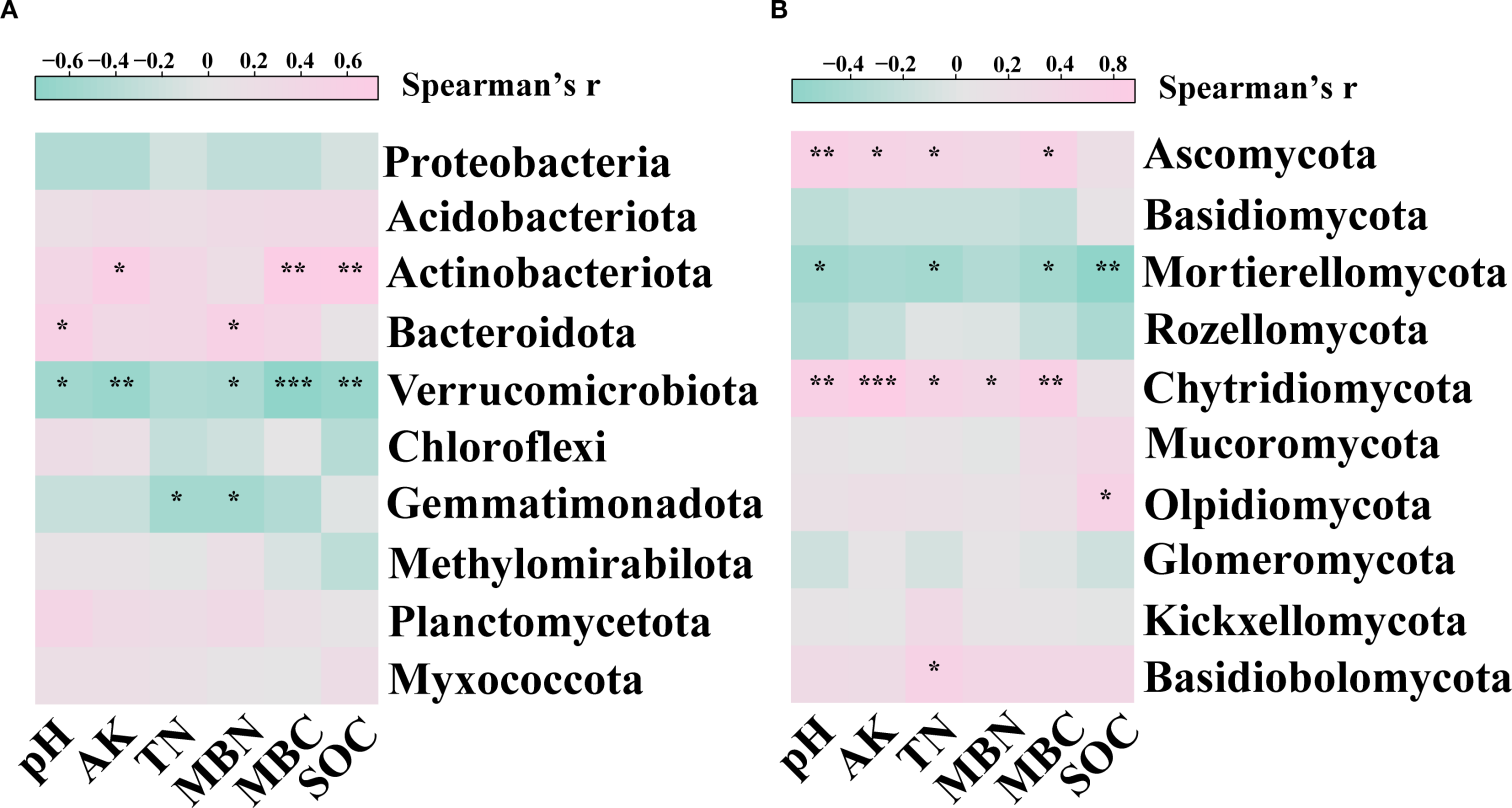
Spearman correlations between soil properties and relative abundance of dominant **(A)** bacterial and **(B)** fungi phyla. Pink and green colors indicate positive and negative correlations, respectively. Asterisks denoting the level of significance (*p< 0.05, **p< 0.01, ***p< 0.001).

Spearman’s correlation analysis showed that the relationship between soil properties and the relative abundance of soil microbes. For bacteria, the relative abundance of Actinobacteriota showed a significant positive correlation (*p*< 0.05) with AK, MBC and SOC; the relative abundance of Bacteroidota showed a significant positive correlation (*p*< 0.05) with pH and MBN; the relative abundance of Verrucomicrobiota showed a significant negative correlation (*p*< 0.05) with pH, AK, MBN, MBC and SOC; the relative abundance of Gemmatimonadota showed a significant negative correlation (*p*< 0.05) with TN and MBN (**Figure 4A**). For fungi, the relative abundance of Ascomycota showed a significant positive correlation (*p*< 0.05) with pH, AK, TN, and MBC; the relative abundance of Mortierellomycota showed a significant negative correlation (*p*< 0.05) with pH, TN, MBC and SOC; the relative abundance of Chytridiomycota showed a significant positive correlation (*p*< 0.05) with pH, AK, TN, MBN and MBC; the relative abundance of Olpidiomycota showed a significant positive correlation (*p*< 0.05) with SOC; the relative abundance of Basidiobolomycota showed a significant positive correlation (*p*< 0.05) with TN (**Figure 4B**).

**Figure 5:**
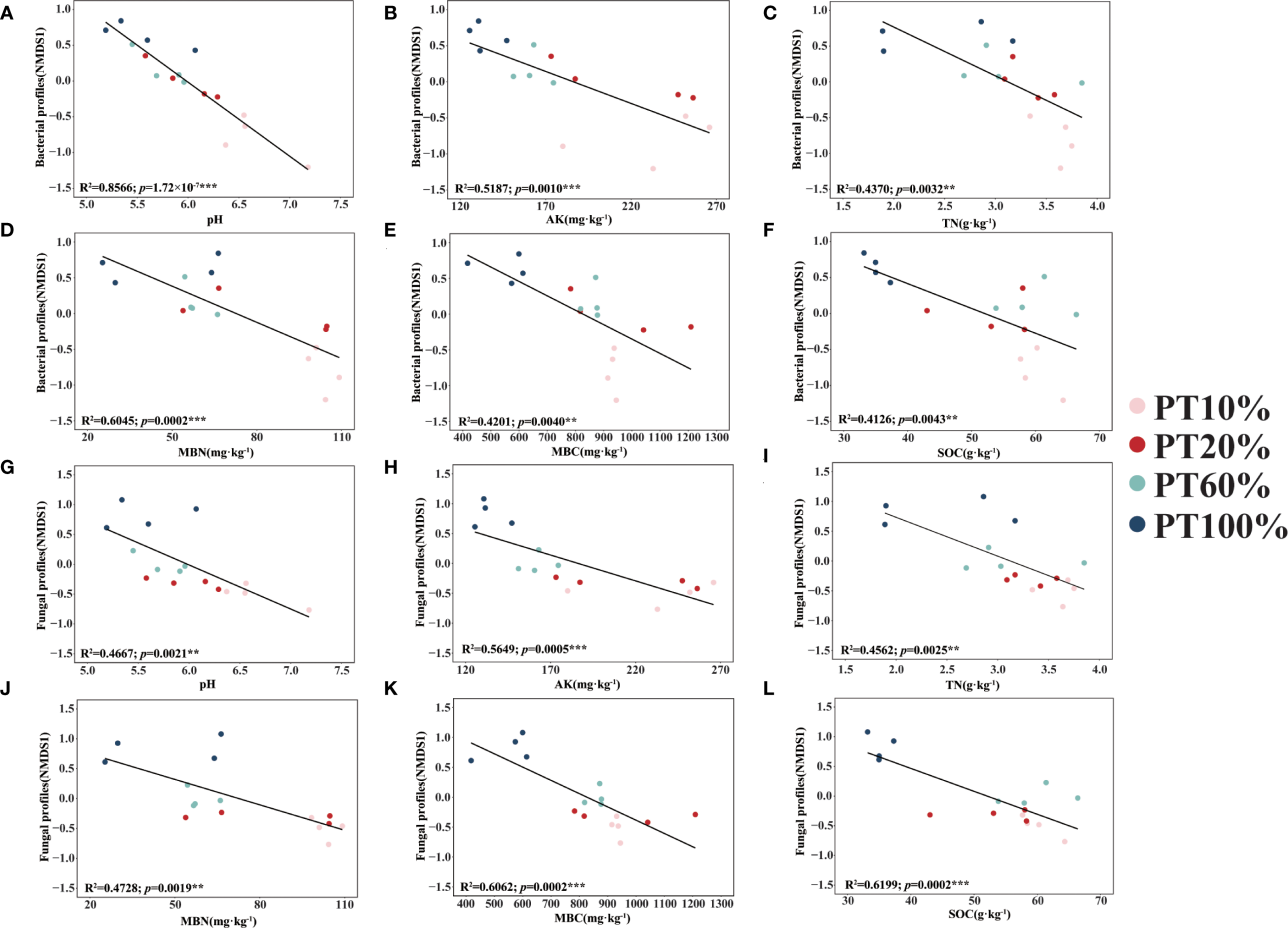
Relationships between soil properties and soil microbiological diversity. **(A–F)** shows the relationships between soil properties (pH, AK, TN, MBN, MBC and SOC) and the first NMDS axis (NMDS 1) of the soil bacterial community, respectively. **(G–L)** shows the relationships between soil properties (pH, AK, TN, MBN, MBC and SOC) and the first NMDS axis (NMDS 1) of the soil fungal community, respectively. Asterisks denoting the level of significance (**p< 0.01, ***p< 0.001).

Soil microbiological β-diversity (NMDS1) was affected by pH (**Figures 5A, G**), AK (**Figures 5B, H**), TN (**Figures 5C, I**), MBN (**Figures 5D, J**), MBC (**Figures 5E, K**) and SOC (**Figures 5F, L**). Between different forests, soil bacterial β-diversity was most affected by pH (R^2^ = 0.8566, *p*< 0.001), in contrast to soil fungal β-diversity (NMDS1), which was least affected by pH (R^2^ = 0.4667, *p*< 0.01). Soil bacterial β-diversity (R^2^ = 0.6045, *p*< 0.001) was more affected by MBN than soil fungal β-diversity (R^2^ = 0.4728, *p*< 0.01), except that soil bacterial β-diversity was less affected by AK, TN, MBC and SOC than soil fungal β-diversity.

**Figure 6:**
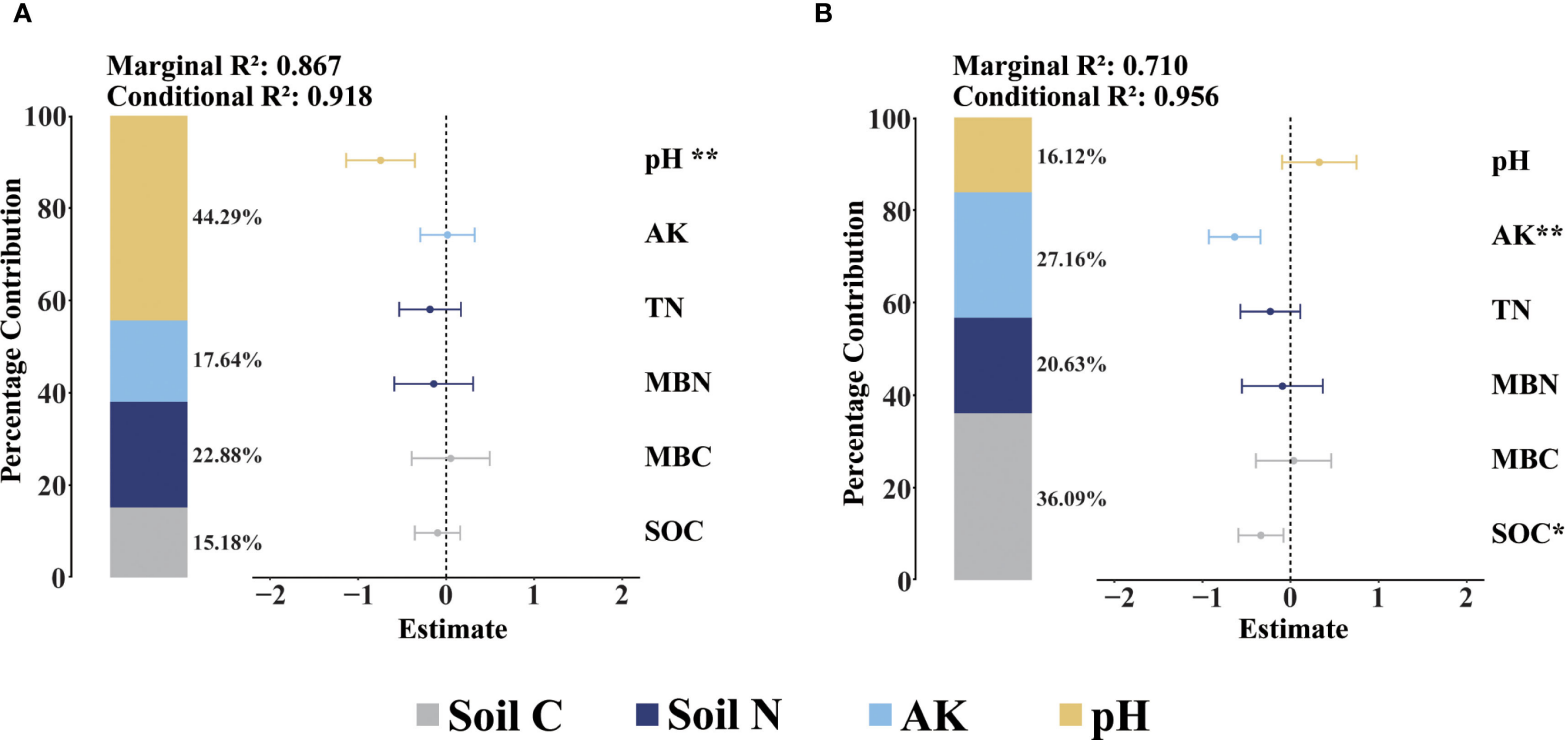
Averaged models for soil microbiological community composition. **(A)** is the average model for soil bacteria, **(B)** is the average model for soil fungi. Parameter estimates and variance explained average models for soil bacteria and fungi. Parameters are classified into four groups: soil C, soil N, AK and pH. Asterisks denoting the level of significance (*p< 0.05, **p< 0.01).

Detailed assessment of the separate GLMM of microorganisms showed that the combination of soil C, soil N, AK and pH explained the variation in bacterial and fungal β-diversity well (Marginal R^2^ > 0.700 and Conditional R^2^ > 0.900). pH was the dominant driver of the variation in bacterial β-diversity and accounted for 44.29% of the fixed effect of bacterial β-diversity (**Figure 6A**). Soil C was the dominant driver of fungal β-diversity change, accounting for 36.09% of the fungal β-diversity fixed effect (**Figure 6B**).

**Figure 7:**
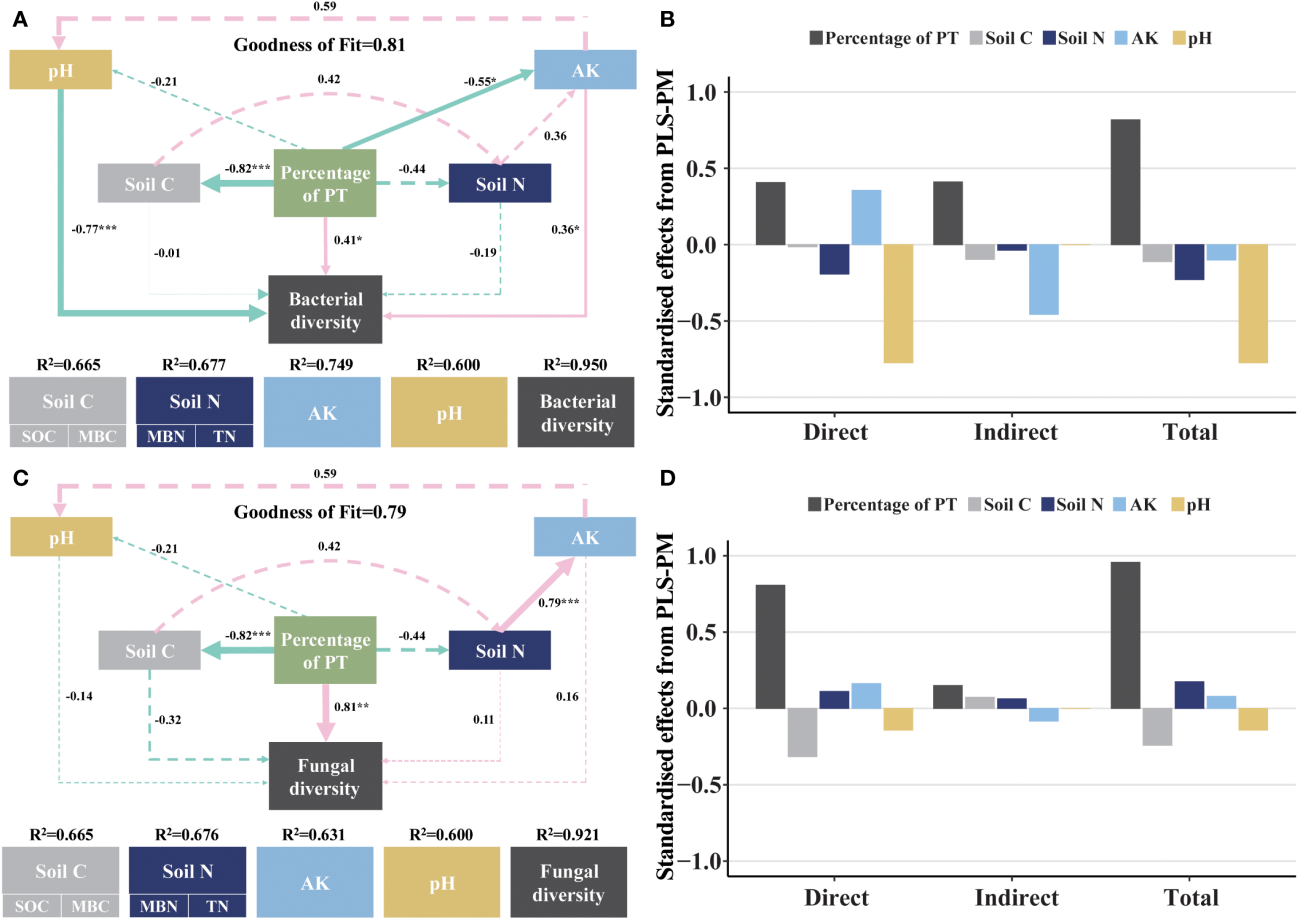
Relationships between soil properties and soil microbiological community composition. **(A)** is the PLS-PM plot of the effects of soil C, soil N, AK and pH on soil bacterial diversity. **(C)** is the PLS-PM plot of the effects of soil C, soil N, AK and pH on soil fungal diversity. Pink solid arrows indicate significant positive effects, green solid arrows indicate significant negative effects, and dashed arrows indicate non-significant effects. The model was evaluated using the goodness-of-fit (GoF) statistic, which is a measure of overall predictive performance. The GoF above the cutoff values of 0.1, 0.25, and 0.36 were classified as weak, moderate, and strong, respectively. **(B, D)** are the standardized direct, indirect, and total effects of each predictor on soil bacterial and fungal composition in the PLS-PM used. Asterisks denoting the level of significance (*p< 0.05, **p< 0.01, ***p< 0.001).

PLS-PM revealed complex interactions between percentage of PT, soil C, soil N, AK, pH and microbiological diversity. For bacteria (GoF = 0.81) (**Figures 7A, B**), percentage of PT had a significant negative effect on soil C (SOC, MBC) (r = -0.82, *p*< 0.001) and AK (r = -0.55, *p*< 0.05) but a positive effect on soil bacterial diversity (r = 0.41, *p*< 0.05). AK positively influenced bacterial diversity (r = 0.36, *p*< 0.05). pH negatively influenced bacterial diversity (r = -0.77, *p*< 0.001). For fungal (GoF = 0.79) (**Figures 7C, D**), percentage of PT had a significant negative effect on soil C (SOC, MBC) (r = -0.82, *p*< 0.001) but a positive effect on soil fungal diversity (r = 0.81, *p*< 0.01). Soil N positively influenced bacterial diversity (r = 0.79, *p*< 0.001).

## 4 Discussion

### 4.1 Effects of different percentages of *P. tabuliformis* planting on the composition of soil microorganisms

Soil properties are sensitive to changes in the proportion of forests planted with *P. tabuliformis*. Among the four forests, soil contents of TN, MBN, AK and SOC were highest in PT10% and lowest in PT100% (**Table 2**). PLS-PM modelling showed that the proportion of forests planted with *P. tabuliformis* had effects on soil C and AK. SOC content was significantly higher in forests with less than 60% *P. tabuliformis* planting than in PT100%, suggesting that low numbers of *P. tabuliformis* plantings can significantly enhance the carbon sequestration capacity of soil. This may be due to the fact that, as the proportion of *P. tabuliformis* planted increases, the proportion of other tree species planted in the forest decreases, the composition of tree species in the forest gradually becomes homogeneous, the understory vegetation is sparse, and the root system and apoplastic material are reduced accordingly, so that the content of SOC that is transformed through decomposition is reduced (Lange et al., 2015; Zarafshar et al., 2020). The accumulation of soil C (MBC, SOC) in the soil provided a more diverse nutrient pool for the growth and development of microorganisms (Hou et al., 2021; Li et al., 2025), thereby increasing the abundance of dominant microbiological communities in the soil (Ji et al., 2014; Chen et al., 2024). The significant positive correlation between the soil C (MBC and SOC) contents and the relative abundance of Actinobacteriota in soil was attributed to the important contribution of Actinobacteriota in increasing soil nutrients and degrading difficult-to-degrade organic matter (Li et al., 2021), which can be involved in carbon sequestration and organic matter cycling (Chen et al., 2024). The results of the study showed that soil AK content decreased with an increase in the proportion of forests planted with *P. tabuliformis*. Coniferous leaves are rich in lignin, and decay of apoplastic material in the forest can degrade lignin in apoplastic coniferous leaves in the soil, thereby releasing nutrients and organic matter (Chen et al., 2017). PT100% is very homogeneous, its apoplastic composition is also relatively simple, and a large amount of lignin in the soil is not degraded, and the soil organic matter content is low. Basidiomycota mainly decomposes lignocellulose (Lundell et al., 2010). Basidiomycota did not show a significant correlation with soil properties, even though it is also a common dominant phylum in all four forest types, a phenomenon that suggests that Basidiomycota is well adapted to different forest community compositions (Wu et al., 2021). Ascomycota is significantly and positively correlated with pH, AK, TN, and MBC. The relative abundance of Ascomycota was close to 50% of the total relative abundance of all fungi in the soil in each of the four forests with different percentages of *P. tabuliformis* plantings. Ascomycota is key decomposers in soil ecosystems (Ma et al., 2013). The results of the study showed that soil TN content and the relative abundance of Ascomycetes decreased gradually with the percentage of *P. tabuliformis* plantings. This trend that indicates the positive impact of Ascomycota on host plants under conditions of high nutrient availability as reported (Leff et al., 2015). At the same time, Ascomycota is well adapted to poor nutrient environments (Sterkenburg et al., 2015), it is effective in degrading organic residues in soil (Hartshornea et al., 2009), and breaks down waste into nutrients that can be taken up by microorganisms (Purahong et al., 2016). Although Ascomycota is better suited to survive in near-neutral soils, the growth and development of Ascomycota was relatively inhibited in near-neutral soils due to the more moderate soil pH and the successive colonization of fungi other than Ascomycota.

### 4.2 Effect of different proportions of *P. tabuliformis* planting on the diversity of soil microorganisms

NMDS analyses revealed significant differences in the β-diversity of soil bacteria and fungi among the four forests, indicating that changes in the proportion of forests planted with *P. tabuliformis* had a significant effect on the β-diversity of soil microorganisms (**Figure 2E, F**). The microbiological co-occurrence network highlighted intricate interactions among soil microbiological communities. All networks displayed modular structures (Modularity > 0.4) (**Figure 3**) (Xue et al., 2017). Although the dominant phyla of soil bacteria and fungi were consistent across four forests, their relative abundance varied significantly. For bacteria, total relative abundance of dominant microbiological phyla was lowest in PT100% soils. pH was key predictor of bacterial diversity (**Figure 6A**), the significant negative correlation between pH and the relative abundance of Verrucomicrobiota suggests that the relative abundance of Verrucomicrobiota increases at low pH, which is due to the relatively narrow growth tolerance of most microbiological taxa (Xue et al., 2017). PLS-PM analyses integrated soil C, soil N, AK, pH and soil microbiological diversity, and elucidated the interrelationships among these components (**Figure 7**) (Zhong et al., 2020; Li et al., 2021). In this study, percentage of PT was associated with changes in soil bacterial diversity. Percentage of PT was found to influence the AK, and changes in AK was an important factor (Zarafshar et al., 2020) affecting the soil bacterial diversity (Lange et al., 2015). pH indirectly affects soil bacterial diversity. The relative abundance of Acidobacteriota was lower in PT100% than in other three forests, and the pH of the PT100% was the most acidic, suggesting that excessive planting of *P. tabuliformis* in forests tends to lead to soil acidification and promotes the growth and development of Acidobacteriota. For fungi, AK and SOC were key predictors of fungi diversity (**Figure 6B**). PLS-PM analyses showed that the percentage of PT was associated with changes in soil fungal diversity. Changes in the proportion of *P. tabuliformis* plantings had a smaller effect on bacterial diversity than fungal diversity, due to the fact that bacteria can metabolize a wider range of compounds, and are therefore more adaptable to environmental change and have a more stable community structure than fungal communities (Uroz et al., 2016; Wang et al., 2019; Cheng et al., 2023).

## 5 Conclusion

This study elucidated the important role of changes in the percentage of planting of *P. tabuliformis* in improving soil nutrients and regulating the composition of soil microbiological communities by analyzing soil properties and soil microorganisms in forests with different percentages of planting of *P. tabuliformis*. The forests with the highest percentage of *P. tabuliformis* planting (PT100%) had the lowest levels of multiple soil nutrients. As the proportion of *P. tabuliformis* planted in the forest decreased, the pH of the soil in the forest improved, and the soil pH gradually changed from acidic to neutral. Soil microbiological community composition was more stable in forests with lower percentages of *P. tabuliformis* plantings (PT20%). Overall, a reduction in the proportion of *P. tabuliformis* planted, accompanied by complementary planting of broadleaf species, could lead to a more stable soil microbiological community composition in forest ecosystems. However, reductions in the proportion of *P. tabuliformis* plantings may alter the characteristics and abundance of co-occurring broadleaf species, which may independently drive the soil environment and microbial responses of the soils in which they are found, given that the litter traits, rooting patterns, and mycorrhizal types of these broadleaf species are markedly different from those of *P. tabuliformis*. Therefore, relevant investigations of broadleaf species composition should be included in subsequent studies.

## Data Availability

The datasets presented in this study can be found in online repositories. The names of the repository/repositories and accession number(s) can be found below: DOI:10.17632/fr36rc4f23.1.
