# Supplementary material for: Reducing the proportion of Pinus tabuliformis planted in mixed coniferous-broad forest stabilizes the microbiological community composition
Source: Front Plant Sci. 2025 Sep 15;16:1646980. doi: 10.3389/fpls.2025.1646980 (PMC12477766; doi:10.3389/fpls.2025.1646980)
Supplement: Supplementary file 1 [file Supplementaryfile1.docx]

Supplementary Material

TABLE S1 Composition of dominant understory plants in different percentages of *Pinus tabuliformis* planting.

| Sample  plot  type | Shrub | | | Herb | | |
| --- | --- | --- | --- | --- | --- | --- |
|  | Species | Relative  dominance/coverage | Importance  value | Species | Relative  dominance/coverage | Importance  value |
| PT10% | Fsu | 0.473 | 29.455 | Cla | 0.220 | 14.447 |
|  | Cma | 0.037 | 10.161 | Mro | 0.154 | 12.773 |
|  | Str | 0.061 | 6.971 | Sca | 0.121 | 9.396 |
| PT20% | Fsu | 0.258 | 17.711 | Cla | 0.392 | 17.324 |
|  | Cmu | 0.205 | 10.973 | Cfl | 0.201 | 13.724 |
|  | Sst | 0.077 | 6.441 | Pum | 0.072 | 10.521 |
| PT60% | Cmu | 0.461 | 26.185 | Cla | 0.336 | 32.339 |
|  | Fsu | 0.331 | 23.771 | Mro | 0.480 | 26.700 |
|  | Sou | 0.040 | 7.100 | Sca | 0.136 | 7.075 |
| PT100% | Fsu | 0.191 | 14.531 | Cla | 0.517 | 31.245 |
|  | Rsa | 0.144 | 9.840 | Pum | 0.310 | 12.337 |
|  | Pin | 0.203 | 9.559 | Tki | 0.110 | 10.254 |

Note: Cma, *Campylotropis macrocarpa*; Cmu, *Cotoneaster multiflorus*; Fsu, *Forsythia suspensa*; Pin, *Philadelphus incanus*; Rsa, *Rubus sachalinensis*; Sou, *Spiraea ouensanensis*; Sst, *Smilax stans*; Str, *Spiraea trilobata*; Cfl, *Clematis florida*; Cla, *Carex lancifolia*; Mro, *Melampyrum roseum*; Pum, *Phlomoides umbrosa*; Sca, *Stipa capillata*; Tki, *Trichosanthes kirilowii*.


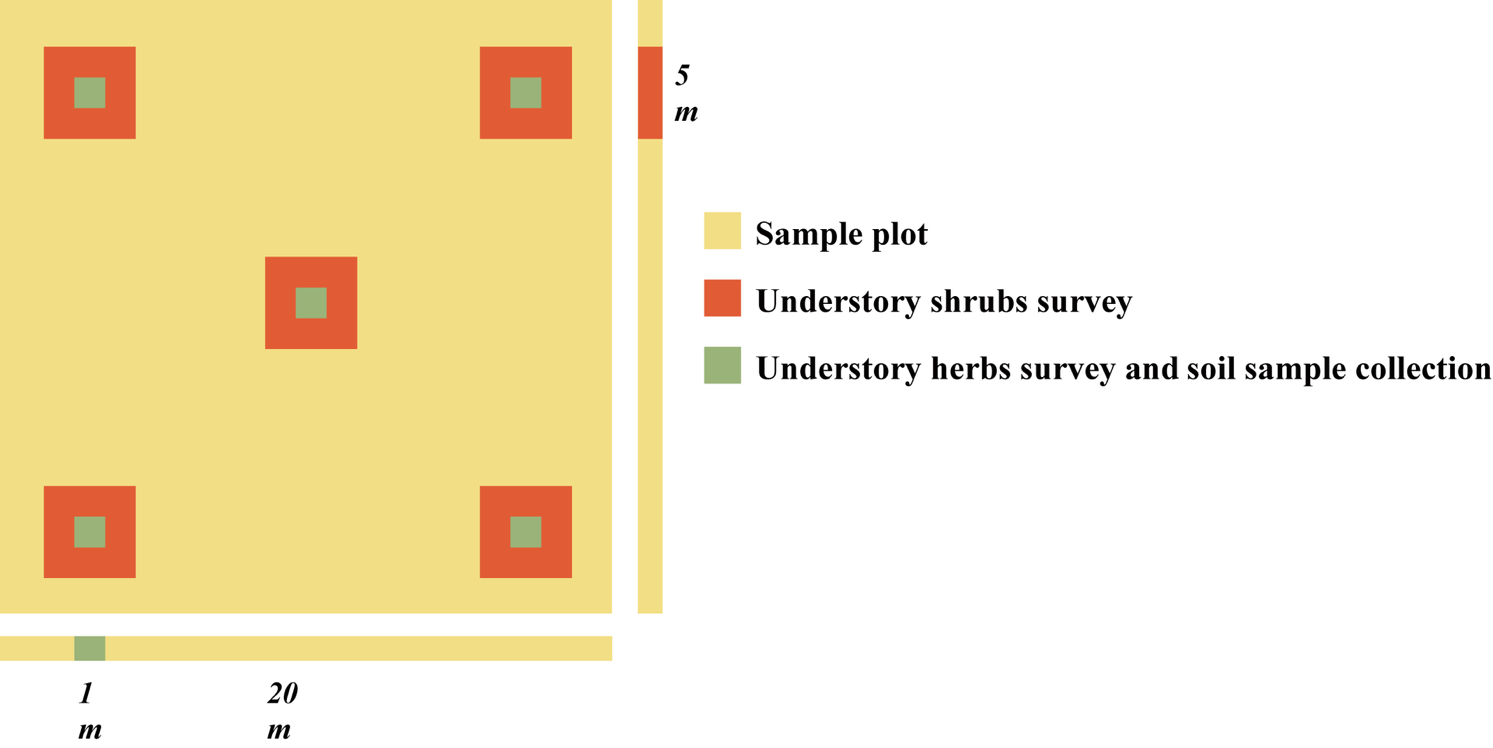


**FIGURE S1** Sample site diagram for understory plant survey and soil sample collection.
